# Nursing intervention in women who developed lymphedema after undergoing a modified radical mastectomy: a pre-experimental study

**DOI:** 10.3332/ecancer.2018.827

**Published:** 2018-04-19

**Authors:** Gloria Daniela de la Borbolla Martínez, Martha Elena Huitzache Martínez, Nicolás Padilla Raygoza

**Affiliations:** 1 School of Nursing, University of Colima, Colima 28040, Mexico; 2 Department of Nursing and Obstetrics, Division of Health Sciences and Engineering, Campus Celaya Salvatierra, University of Guanajuato, Guanajuato 38060, Mexico

**Keywords:** self-care demand, breast cancer, lymphedema, mastectomy

## Abstract

The aim of this study was to analyse the effect of a nursing intervention in increasing the therapeutic self-care demand in patients with breast cancer-related lymphedema after a mastectomy. The pre-experiment was carried out on women in the State Cancer Institute in Colima, Mexico. Thirty women who had undergone a mastectomy were included in this study. Each woman received a nursing intervention to increase her knowledge, skills and motivation, which are the components of therapeutic self-care demand. Students’ *t* and *P* values were calculated for paired means, and the McNemar X^2^ test was performed for paired categorical variables. It was found that Students’ *t* values for therapeutic self-care demand, knowledge, skills and motivation were significant (all with *P* = 0.00001). It was also found that McNemar *X *^2^ values for the same criteria paired categorically were significant (*P* = 0.0002, *P* = 0.003, *P*= 0.0002, *P* = 0.00001, respectively). It was concluded that the nursing intervention was effective in increasing the therapeutic self-care demand of patients with post-mastectomy breast cancer.

## Background

Post-mastectomy upper limb oedema has been described as ‘the most unpleasant and frequent nonlethal complication, and the only one with a loss of function in the affected upper limb’ [1].

It is caused by inevitable damage to the lymphatic collecting vessels and/or by surgically removing the axillary lymph nodes. Generally, this type of oedema produces a feeling of heaviness or tightness, decreased muscle strength, restricted joint movements, and can sometimes cause pain in the entire upper limb and back [2].

Breast cancer and its post-surgical side effects have physical, emotional, behavioural, spiritual, social and familial consequences for women and their families. These effects can affect women from the point of diagnosis to treatment and rehabilitation.

Breast cancer is the most common cancer in women worldwide and has become a public health problem, with mortality continuing to rise in Mexico. Breast cancer causes more deaths in Mexico than any other malignant neoplasia, mainly affecting women over 25 years old [3].

The overall incidence of arm oedema after breast cancer treatment is 26% [4]. In 1997, Mortimer reported that the incidence of lymphedema in 1,151 women treated with radiation therapy rose from 23% at 0–2 years after treatment to 45% at 15 years or more since treatment. In patients treated with surgery alone, incidence increased from 20% at 0–2 years to 30% after 15 years or more since treatment [5].

DiSipio *et al* [6] reported that lymphedema was present in approximately 20% of women who had undergone node resection. This percentage increased to 21% after the removal of axillary lymph nodes, and 38% after post-mastectomy radiation therapy.

The American Cancer Society reported that 10–35% of patients who underwent axillary lymph node dissection and/or radiation therapy are likely to develop some degree of lymphedema [7]. The Spanish Cancer Association states that breast cancer patients treated with axillary lymph node dissection have up to a 10% risk of developing lymphedema. That risk increases to 20–25% in patients who receive axillary radiation therapy as well as having surgery [8].

It is estimated that one in four women with breast cancer will develop lymphedema in the affected arm, thus affecting their quality of life. Lymphedema is a major problem because of the number of women who need treatment and the resulting costs to the healthcare system [9].

There are two forms of lymphedema, early and late. Early-onset lymphedema occurs up to 2 months after surgery and is usually temporary. Late-onset lymphedema can occur at any time after 6 months of initial treatment and is often progressive [10]. Both forms of lymphedema impair mental and physical function and cause pain [11]. Lymphedema is one of the most serious sequelae that affects the quality of life of women who have surgery for breast cancer [12]. It has been described as the most distressing long-term complication of breast surgery [13].

Raising awareness of patient self-care activities is crucial for preventing and controlling lymphedema. This means informing patients of the extent or lack of improvement based on their participation, combined with the support of nursing staff.

Orem’s theory emphasises that performing self-care requires deliberate, intentional and calculated action conditioned by an individual’s knowledge and skill set. The theory rests on the premise that individuals know when they need help and are thus are aware of the specific actions they need to perform. The main assumption of Orem’s theory is that ‘self-care is not innate’, but rather a learnt behaviour. Self-care thus grows and develops as people grow and develop, influenced by their surrounding environment [14].

We could not find any published studies online of self-care in women with post-mastectomy lymphedema, and decided to carry out this study. Whilst searching, we found reports of self-care in breast cancer and many other conditions, including high blood pressure, kidney disease and type 2 diabetes.

The aim of this study was to test the effect of a nursing intervention in increasing the therapeutic self-care demand of women who underwent a mastectomy in Colima, Mexico.

## Methods

The study used a social science model of a pre-experimental, longitudinal, prospective design. The study was carried out in the Colima State Cancer Institute in Colima, Mexico (IEC in Spanish), on a population of 30 women with post-modified radical mastectomy lymphedema (PMRM).

There was no sampling as the population was small, and it was decided to include all the women who met the selection criteria and agreed to participate. This approach ensured that participants would receive the best treatment possible.

## Sample size

A median difference of 15 was assumed, with a standard deviation in the therapeutic self-care demand scores of 6 before the intervention and 7 after. The resulting minimum sample size was three participants with results before and after the intervention (Epidat 4.1, 2013, Xunta de Galicia, World Health Organization, CES University)

## Participant recruitment

Inclusion criteria were the following: women aged 18 or over who underwent a modified radical mastectomy, at any clinical stage of lymphedema, registered at the IEC and had agreed in writing to participate voluntarily in the investigation by signing the informed consent document.

Exclusion criteria were: women who did not develop PMRM lymphedema and women who did not agree to participate.

## Variables

Sociodemographic variables were age, education level, occupation, marital status, time since mastectomy, and stage of cancer at diagnosis. The independent variable was the nursing intervention, defined as ‘Any treatment, based on clinical judgement and knowledge that a nurse performs to enhance client outcomes’ [15]. The working definition is: actions/interventions that a nurse performs to educate and advise patients on treating and preventing PMRM lymphedema, so that patients make the best decisions in increasing their self-care and therefore the therapeutic self-care demands. The study focused on three components: knowledge, skills and motivation. Knowledge refers to knowing the therapeutic self-care demands of the arm with PMRM lymphedema. Skills refer to the skills needed to carry out the therapeutic self-care demands of the arm with PMRM lymphedema. Last, where motivation is the development of a positive attitude. The educational support programme and nursing interventions were used in an attempt to improve the overall quality of life of patients diagnosed with lymphedema.

A registered nurse who is an expert in the field carried out this programme, spending an hour with each patient.

The intervention was based on evidence supporting forms of treatment that the post-mastectomy patients should use to prevent or control lymphedema. Those treatments include manual lymphatic drainage (MLD), compression bandages, patient education and skin care. The entire intervention was focused on the knowledge of MLD, compression bandages and skin care. Participants demonstrated that they knew how to apply these treatments correctly (skills) and the study attempted to measure their motivation to apply them correctly.

The dependent variable was the self-care demand, or the actions or activities that a patient with lymphedema performs in a given period of time. Those actions or activities serve to satisfy the self-care needs of patients and thus improve therapeutic self-care demands. Performance was measured using the therapeutic demands questionnaire. Scoring ranged from 0 to 45 points: 36 to 45 points for adequate therapeutic self-care demands and <36 for inadequate. There were three scoring components: knowledge, with 20–25 points for adequate knowledge and <19 for inadequate; skills, with 10–12 points for adequate skills and <9 for inadequate; and motivation, with 6–8 points for adequate motivation and <6 for inadequate. Scores were summarised using frequencies and percentages.

## Evaluation tools

A tool was created specifically to measure the therapeutic self-care demands of women with PMRM lymphedema, and was used to identify patient knowledge, skills and motivation. The tool was pilot tested, and achieved a reliability rating of 0.96 using Kendall’s test. To ensure the tool was valid, criteria were established based on evaluation, composition and length, as well as content when relevant. A group of experts was selected, consisting of five nurses with extensive experience both in caring for breast cancer patients and research. Those five nurses were given the evaluation tool with the 45 items and criteria to evaluate. They determined whether the sets of items were representative of and sufficient for each of the categories that the tool evaluates. The tool consists of three parts, and the dichotomous measurement scale is used in each one, where 1 = yes and 0 = no. The maximum total of points was thus 45 for good therapeutic self-care demand.

## Procedures

Once the conduct of the study was authorised, a meeting was held with potential participants to explain study aims. After their questions were answered, participants were asked to sign the informed consent document. Sociodemographic data and the therapeutic self-care demands questionnaire were collected. The nursing intervention was then delivered over 30 days, and the post-intervention self-care demands questionnaire was then completed.

### Statistical analysis

Descriptive statistics were applied to categorical and quantitative sociodemographic variables. To test the hypothesis, the difference in the pre- and post-intervention self-care capacity scores and their individual components were calculated. Those results were used to perform a Student’s *t* test for paired means and to calculate a *P* value. The McNemar’s *X*^2^ value for paired categorical variables and the *P* value were also calculated. To demonstrate statistical significance of the results, the value of *P* was set to 0.05. Statistical analysis was performed using STATA 13.0® (StataCorp., College Station, Texas, USA).

## Results

The sample consisted of 30 women studied at the State Cancer Institute. Most of the women were married housewives with a primary school education, were 12–36 months post-mastectomy, and had stage III breast cancer at diagnosis (Table 1).

The therapeutic self-care demands were classified by total score, knowledge score, skills score and motivation score. Patient therapeutic self-care demand scores were statistically significant when comparing the median of the differences (before and after the nursing intervention) (*P* = 0.0001) (Table 2).

Pre- and post-intervention therapeutic self-care demands, knowledge, skills and motivation were statistically significant (*P* =.0002, *P*=.003, *P*=.0002 and *P*=.0001, respectively) (Table 3).

## Discussion

The study included all members of the population, avoiding sampling bias. There were no drop-outs during follow-up, giving the study a major advantage. There was 100% attendance at the intervention sessions.

The small study population was a disadvantage, but allowed the nursing intervention to be much more personal. The study lacked a control group, and results were neither compared nor controlled.

It has been reported that patients do not receive basic information on the risk of lymphedema after breast cancer surgery and/or radiation therapy for breast cancer. It has also been reported that general patient knowledge of PMRM lymphedema risk factors and prevention strategies is poor [16].

Patients in this study had not received any kind of information that would allow them to acquire therapeutic self-care demands prior to the nursing intervention. Post-intervention results showed that in 56.6% of the women there was a change in therapeutic self-care demands (Table 3).

Dorothea Orem emphasises the importance of knowing and deliberately performing healthcare actions. Her self-care deficit theory states that therapeutic self-care demands are the number and type of actions or activities that a person performs or should perform in a given amount of time to meet self-care needs. A person meets those needs by developing the ability to care for herself until reaching maturity.

Orem also explains how a person suffering from a disease needs the support of nursing staff trained to help patients develop self-care capacity. Nursing and the actions of nurses thus focus mainly on providing physical care for others. This includes using information to guide or direct patients and providing physical and/or psychological support. Nurses engage in dialogue to achieve co-operation and understanding with patients, helping them meet self-care needs. Through these actions, nurses provide a suitable environment for patients to mitigate limitations and develop their skills. Last, nurses teach basic methods and actions that help patient meet their self-care demands.

The benefits of this research and the results obtained in this study show that implementing the educational support programme and the nursing intervention resulted in a significant difference in therapeutic self-care demands (p < 0.05) (Table 3) improving the quality of life in women with breast cancer, for whom lymphedema disrupts daily work and social activities and has physical and psychological consequences. It is also known that nursing interventions to address lymphedema will increase knowledge, skill and motivation; and patients with lymphedema will receive the appropriate treatment. These results show patients that although this is an evolutionary process in the natural history of PMRM lymphedema, treatment is available [17].

There is currently no standard, shared, multidisciplinary intervention protocol for lymphedema. There is also a lack of training and information to educate patients about what lymphedema is how to prevent it and how to treat it. These problems result from the invisibility of lymphedema to healthcare professionals, patients, and their social circles. This study therefore involved only work with interventions that could be carried out by nurses.

This study demonstrated that nursing actions meet community expectations and health policies by advancing the health promotion strategy of the World Health Organization. The nursing intervention in this study was of great importance to patients with PMRM lymphedema, as it is low-cost.

Similarly, the therapeutic self-care demands of women with PMRM lymphedema after the nursing intervention contributed increase self-care requirements. That development ensures a better quality of life during and after treatment.

Mexico has an official Mexican standard, NOM-041-SSA2-2002, for breast cancer prevention, diagnosis, treatment, control and epidemiological monitoring; a National Epidemiological Monitoring and Disease Control Centre; and a Ministry of Health Action Programme: Breast Cancer [18, 19]. A review of these institutions and policies shows that there is no strategy or programme to prevent and treat lymphedema in breast cancer patients.

The Mexican Federal Official Gazette states that all patients undergoing treatment for breast cancer should be evaluated to determine the type of rehabilitation they need. Patient rehabilitation should, based on patient needs, include: physiotherapy, the use of prosthesis to keep shoulders symmetrical, breast reconstruction and lymphedema treatment [20].

Timeus and Padilla [21] found that complete decongestive therapy markedly reduces the circumference of all areas of the affected upper limb, with statistically significant differences (*p* < 0.05). The participants in the Colima demonstrated the knowledge and skills to perform lymphatic drainage (Table 3).

Liao *et al* [22] found the compression bandage useful in controlling post-mastectomy lymphedema. Participants in this study demonstrated skills in using the compression bandage included in the therapeutic self-care demand skills (Table 3).

Stubblefield reports an insufficient use of physical therapy and rehabilitation services in breast cancer patients. That insufficient use is mainly due to a lack of knowledge among patients and treating physicians, and limited access to the services [23]. This finding shows the importance of nursing professionals in raising awareness of the self-care demands of post-mastectomy patients.

## Conclusion

The nursing intervention used to increase the three components of the therapeutic self-care demand—knowledge, skills and motivation—proved effective. This intervention will have a positive effect on the well-being of post-mastectomy patients and improve their quality of life.

## Conflicts of interest

The authors declare that they have no conflicts of interest.

## Authors’ contributions

DBM designed the protocol and drafted the article. MEHM searched the literature and participated in the design of the protocol. NPR carried out the statistical analysis and participated in drafting the report. The authors approved the final version of the manuscript.

## Figures and Tables

**Table 1.** Categorical sociodemographic characteristics of the sample participants (*n* = 30).

Variable	f%
**Education level** None Primary school Secondary school Sixth form college University	5–16.67 11–36.67 9–30.00 4–13.33 1–3.33
**Occupation** Housewife Student Employed Unemployed Retired	19–63.34 1–3.33 6–20.00 3–10.00 1–3.33
**Marital status** Single Married Divorced Cohabiting	8–26.66 14–46.67 6–20.00 2–6.67
**Time since mastectomy (months)** <6 6–11 12–36 48–84 96–120	2–6.67 6–20.00 17–56.66 2–6.67 3–10.00
**Breast cancer stage** Unknown I II III IV	3–10.00 4–13.33 7–23.33 15–50.00 1–3.33

**Table 2.** Median difference of the therapeutic self-care demand scores and its components, before and after the intervention (*n* = 30).

Therapeutic self-care demand	Before	After	Median difference ± S	*t*	df	*P* value
Total	12.50	31.47	−18.97±7.36	−14.11	29	0.00001
Knowledge	6.63	16.33	−9.70±4.75	−11.19	29	0.00001
Skills	2.20	8.40	−6.20±2.23	−15.23	29	0.00001
Motivation	3.70	6.73	−3.03±1.47	−11.29	29	0.00001

S: standard deviation

**Table 3.** Therapeutic self-care demands and its components, before and after the intervention (*n* = 30).

**Pre intervention**		**Post-intervention**			
		**TSD** **I A**	**Knowledge** **I A**	**Skills** **I A**	**Motivation** **I A**
	TSD I A	*X *^2^ = 13.00, df = 1, *P* = 0.0002 17 13 0 0			
	Knowledge I A		*X *^2^ = 9.00, df = 1, *P* =.003 21 9 0 0		
	Skills I A			*X *^2^ = 13.00, df = 1, *P* = 0.0002 17 13 0 0	
	Motivation I A				*X *^2^ = 21.00, df = 1, *P* = 0.00001 4 21 0 5

TSD: therapeutic self-care demands; I: inadequate; A: adequate; Df: degrees of freedom
